# Red cell distribution width and mortality in older patients with frailty in the emergency department

**DOI:** 10.1186/s12873-023-00801-1

**Published:** 2023-03-09

**Authors:** Janne Alakare, Kirsi Kemp, Timo Strandberg, Maaret Castrén, Jukka Tolonen, Veli-Pekka Harjola

**Affiliations:** 1 Department of Emergency Medicine and Services, Helsinki University Hospital and University of Helsinki, Helsinki, Finland; 2 Department of Geriatric Acute Care, Espoo Hospital, 2550 02070 City of Espoo, PL Finland; 3 University of Helsinki, Clinicum, and Helsinki University Hospital, Helsinki, Finland; 4 University of Oulu, Center for Life Course Health Research, Oulu, Finland; 5 Department of Internal Medicine, Helsinki University Hospital and University of Helsinki, Helsinki, Finland

**Keywords:** Frailty, Red cell distribution width, Biomarkers, Mortality, Prognostic factor, Emergency departments

## Abstract

**Background:**

The red cell distribution width (RDW) reflects the degree of heterogeneity of red blood cells. Elevated RDW is associated both with frailty and with increased mortality in hospital-admitted patients. In this study we evaluate whether high RDW values are associated with mortality in older emergency department (ED) patients with frailty, and if the association is independent of the degree of frailty.

**Methods:**

We included ED patients with the following criteria: ≥ 75 years of age, Clinical Frailty Scale (CFS) score of 4 to 8, and RDW % measured within 48 h of ED admission. Patients were allocated to six classes by their RDW value: ≤ 13%, 14%, 15%, 16%, 17%, and ≥ 18%. The outcome was death within 30 days of ED admission. Crude and adjusted odds ratios (OR) with 95% confidence intervals (CI) for a one-class increase in RDW for 30-day mortality were calculated via binary logistic regression analysis. Age, gender and CFS score were considered as potential confounders.

**Results:**

A total of 1407 patients (61.2% female), were included. The median age was 85 with an inter-quartile range (IQR) of 80–89, median CFS score 6 (IQR: 5–7), and median RDW 14 (IQR: 13–16). Of the included patients, 71.9% were admitted to hospital wards. A total of 85 patients (6.0%) died during the 30-day follow-up. Mortality rate was associated with RDW increase (*p* for trend < .001). Crude OR for a one-class increase in RDW for 30-day mortality was 1.32 (95% CI: 1.17–1.50, *p* < .001). When adjusted for age, gender and CFS-score, OR of mortality for one-class RDW increase was still 1.32 (95% CI: 1.16–1.50, *p* < .001).

**Conclusion:**

Higher RDW values had a significant association with increased 30-day mortality risk in frail older adults in the ED, and this risk was independent of degree of frailty. RDW is a readily available biomarker for most ED patients. It might be beneficial to include it in risk stratification of older frail ED patients to identify those who could benefit from further diagnostic assessment, targeted interventions, and care planning.

## Background

Red cell distribution width (RDW) is a measure reflecting the degree of heterogeneity of red blood cell size. RDW is calculated by dividing the standard deviation of red blood cell volumes by the mean corpuscular volume (MCV), usually expressed as a percentage value. RDW has traditionally been used for differential diagnosis of anaemia, but, subsequently, RDW elevation has been found to associate with higher short- and long-term increased mortality, both in the general population and in patients with many specific conditions [1–5], such as infections and sepsis [6–8], liver cirrhosis [9, 10], diabetic ketoacidosis [11], trauma [12, 13], acute pancreatitis [14], cardiac diseases [8, 15–19], pulmonary embolism [20], COVID-19, and acute respiratory failure [21–23]. In hospitals, high RDW has been shown to predict poor prognosis among general patients [24], among surgical patients [25], and among patients with critical illness [26, 27]. Older patients in emergency departments (EDs) and hospital wards have an increased risk of mortality if their RDW is elevated [8, 12, 28–30]. Although several mechanisms for this association have been presented [1, 2, 31], defined mechanisms for the association of elevated RDW and increased mortality have not yet been established.

Frailty syndrome, an ageing-related state of vulnerability due to decline in physiological reserves and functions [32], is usually defined either as a clinical phenotype or by calculating accumulated deficits such as diseases, physical and cognitive impairments, psychosocial risks, and geriatric syndromes [33–35]. Frailty is an independent predictor of mortality in patients admitted to emergency departments and hospital wards [36–38]. Elevated RDW has been shown to associate with frailty besides increased mortality of older ED patients [39–41].

Because frailty is related with both increased mortality of older ED patients and elevated RDW, frailty may be a confounder explaining increased mortality of older patients with elevated RDW. We studied whether elevated RDW is a risk predictor in older patients with frailty in the ED, and how frailty stage affects the association between elevated RDW and mortality.

## Methods

This study is a secondary analysis of an observational cohort study in frail older ED patients that was performed in an ED of a teaching hospital in Finland. In the primary study we included patients who were ≥ 75 years of age, had a score between 4 to 9 on the 9-point Clinical Frailty Scale (CFS) [34], and were registered residents of the hospital’s service area. ED visit data were collected between December 11^th^, 2018 and June 7^th^, 2019. The included patients were followed up from electronic health records. Methods for the primary study have been described in detail in our previous article [42].

The clinical laboratory service of the ED routinely gives RDW values (% value as integer) for all blood counts tested. Besides the clinical laboratory service, the ED has point-of-care testing equipment available, which does not provide RDW values. Point-of care testing is typically preferred, if more extensive laboratory testing is not anticipated based on patient’s chief complaint or condition. For the secondary analysis conducted here, those patient visits from the primary study who had the CFS score 4–8 and had RDW tested 0–48 h after ED admission were included. If more than one blood count was drawn from a patient within 48 h of ED admission, the result of the first laboratory test was used for the analysis. Patients who had a CFS score of 9 were excluded because such patients are defined as having a short life expectancy < 6 months, but otherwise not living with severe frailty.

Nonparametric baseline data were presented with interquartile ranges (IQR). The outcome measure was 30-day mortality. Patients were allocated to six classes based on their RDW value: ≤ 13%, 14%, 15%, 16%, 17%, and ≥ 18%. We used same cut-off values as a recent study to enable comparison of our results in frail ED patients to general older adult ED patient population [42]. Mortality rate was calculated for each class. The Cochran–Armitage test for trend was used to test the statistical significance of the trend of increasing mortality with higher RDW values.

Crude and adjusted ORs with 95% confidence intervals (CI) of a one-class increase in RDW for 30-day mortality were calculated. Univariate and multivariate models of binary logistic regression analysis were used for crude and adjusted ORs, respectively. Age, sex, and CFS score were considered as potential confounders and were included in the analysis.

As a sensitivity analysis to assess if categorisation of the RDW values has impact on the results, we performed a regression analysis with RDW as continuous variable. We also performed a sensitivity analysis with haemoglobin as a potential confounder, because haemoglobin level is directly related to red blood cells, like RDW is, and may be associated with mortality.

From clinical perspective, we were interested whether RDW is independent of vital parameters. The National Early Warning Score 2 (NEWS2), a widely used prognostic score based on common vital signs, was included in the baseline data for our previous study [42]. We performed an additional testing by adjusting with the NEWS2 besides other potential confounders used in the regression analysis.

A *p* value of < 0.05 was considered statistically significant. GraphPad Prism software, version 9.4.1 (Graphpad Software LCC) was used for the Cochran–Armitage test. SPSS software, version 28 (IBM) was used for all other statistical analyses.

The primary study which this secondary analysis was based on, was registered at ClinicalTrials.gov on December 20t^h^, 2018, identifier NCT03783234.

## Results

**Fig. 1:**
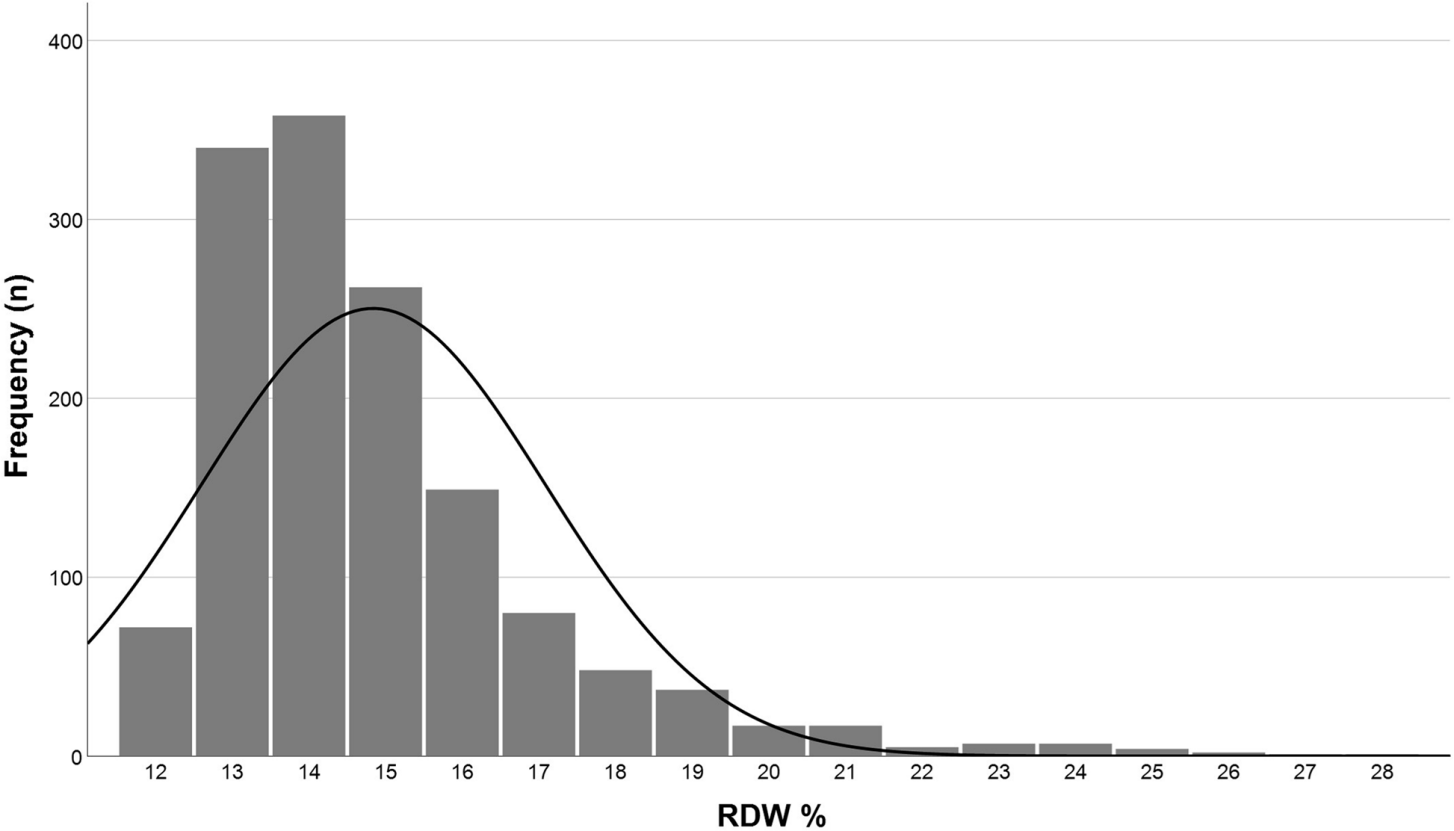
Distribution of RDW % in included patients. Normal distribution marked by black curve. Abbreviation: RDW, red cell distribution width

A total of 1407 (61.2% female) patient visits were included after excluding 294 visits for patients who either had no blood count drawn within 48 h of ED admission or had only point-of-care blood count testing without RDW-values, as well as four cases for patients with dual peak RDW values (due to previous red cell transfusions), and seven cases for patients with a CFS score of 9. Patient characteristics for the analytical sample are presented in Table 1: median age was 85 (IQR: 80–89), median CFS was 6 (IQR: 5–7), and median RDW % was 14 (IQR: 13–16, range: 12–28). Distribution of the RDW % values in the analytical sample are presented in Fig. 1. Of the included patients, 1011 (71.9%) were admitted to hospital wards.

**Table 1:** Patient characteristics

	N	1407
Age	median (IQR)	85 (80–89)
Female	n (%)	861 (61.2)
Hospital admission	n (%)	1011 (71.9)
CFS	median (IQR)	6 (5–7)
CFS: 4	n (%)	282 (20.0)
CFS: 5–6		718 (51.0)
CFS: 7–8		407 (28.9)
RDW %	Median (IQR)	14 (13–16)
RDW ≤ 13%	n (%)	412 (29.3)
RDW 14%		358 (25.4)
RDW 15%		262 (18.6)
RDW 16%		149 (10.6)
RDW 17%		80 (5.7)
RDW ≥ 18%		146 (10.4)

*Abbreviations*: *IQR* Interquartile range, *CFS* Clinical Frailty Scale, *RDW* Red cell distribution width

**Fig. 2:**
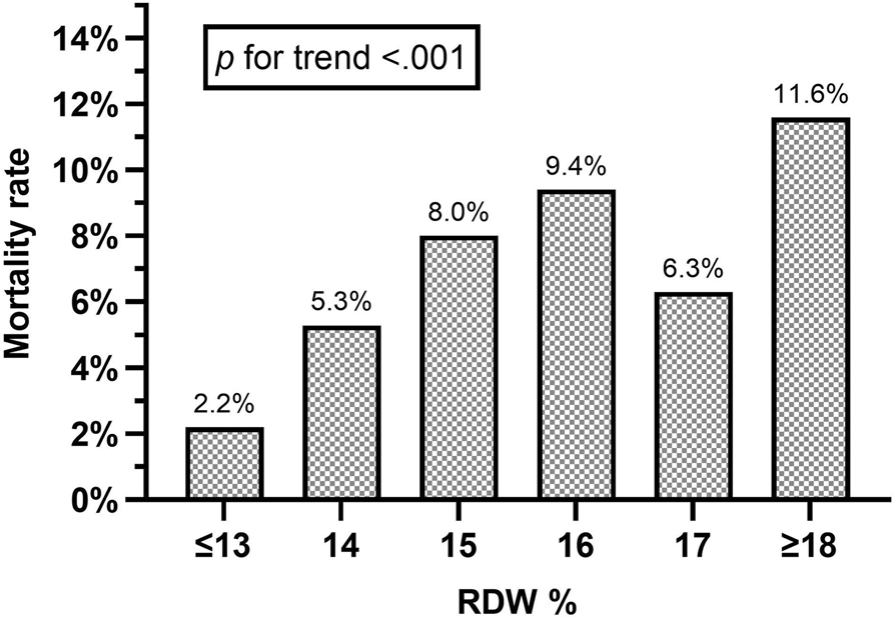
30-day mortality rates in each red cell distribution width category. The Cochran–Armitage test for trend was used to test the statistical significance of the trend. Abbreviation: RDW, red cell distribution width

Follow-up data for 30-day mortality were available for all ED visits. A total of 85 of 1407 (6.0%) of included patients with RDW value available died during the 30-day follow-up. Within 30 days of ED admission, mortality rates were as follows: 9/412 (2.2%) of patients in the RDW ≤ 13% group, 19/358 (5.3%) in the RDW 14% group, 21/262 (8.0%) in the RDW 15% group, 14/149 (9.4%) in the RDW 16% group, 5/80 (6.3%) in the RDW 17% group, and 17/146 (11.6%) in the RDW ≥ 18 group. Mortality rate was significantly higher with an increase in RDW (*p* for trend < 0.001). Mortality rates are presented in Fig. 2. For comparison, 30-day mortality of excluded patients who had no RDW value available was 8/298 (2.7%).

Crude OR of a one-class increase in RDW for 30-day mortality was 1.32 (95% CI: 1.17–1.50, *p* < 0.001). When adjusted for age, sex and CFS score, OR of a one-class increase was still 1.32 (95% CI: 1.16–1.50, *p* < 0.001). Crude and adjusted odds ratios are presented in Table 2.

**Table 2:** Crude and adjusted odds ratios for 30-day mortality

	OR, crude (95% CI)	*p* for crude OR	OR, adjusted (95% CI)	*p* for adjusted OR
RDW ^a^	1.32 (1.17–1.50)	< .001	1.32 (1.16–1.50)	< .001
CFS ^b^	1.47 (1.22–1.78)	< .001	1.43 (1.18–1.73)	< .001
Age ^c^	1.03 (1.00–1.07)	.076	1.04 (1.00–1.08)	.082
Female	0.73 (0.47–1.14)	.169	0.67 (0.42–1.05)	.083

Binary logistic regression was used for the analysis. RDW class, CFS, age, and sex were included in the analysis for adjusted odds ratios

*Abbreviations*: *RDW* red cell distribution width, *CFS* Clinical Frailty Scale, *OR* odds ratio, *CI* confidence interval

^a^one-class increase

^b^one-point increase

^c^one-year increase

In the sensitivity analysis with RDW as a continuous variable the significance of the results were not changed. Crude and adjusted ORs of 1%-unit increase of RDW for 30-day mortality were: 1.15 (95% CI: 1.06–1.24, *p* < 0.001), and 1.15 (95% CI: 1.07–1.25, *p* < 0.001), respectively. The absolute OR values were expectedly lower as scale increased from 6 categorical steps to 16 steps in %-units (range of RDW, 12–28). When haemoglobin level was added as a potential confounder, the adjusted OR of one-class increase in RDW was 1.34 (95% CI: 1.17–1.54, *p* < 0.001), without significant change in results.

When NEWS2 was added as a confounder, the adjusted OR of one-class increase of RDW for 30-day mortality was slightly lower than without it, but still significant: 1.27 (95%: 1.11–1.47, *p* < 0.001).

## Discussion

In this study, increasing RDW was associated with higher 30-day mortality in frail older ED patients. The association remained significant when adjusted for age, gender and CFS score.

This study shows that the association of higher RDW value and increased mortality applies to the frail older population in an acute care setting. The association is independent of CFS score. The mortality rate increase is similar to those rates shown in a recent large cohort study of general hospital-admitted older patients [29], supporting the hypothesis that RDW is independent of frailty as a risk predictor. A small notch in mortality rate was noted in the group of patients with RDW of 17%. However, since the total trend was statistically significant, we interpret this dip to be variation due to limited sample size.

Many mechanisms, both short- and long-term, have been suggested for the association of elevated RDW and increased mortality. Impaired erythropoiesis and shortened red cell survival due to organ dysfunction, metabolic imbalances, and inflammatory reactions may cause alterations in red cell volumes. Oxidative stress and suppression of the erythrocyte lineage due to alterations in neutrophil and thrombocyte production during inflammation in acute conditions are potential contributors. Other possible causes of higher RDW include poor nutrition and erythrocyte fragmentation [1, 2, 31]. In addition, direct causality of high RDW and impaired intravascular haemodynamics, especially with vascular pathologies, has been presented [31]. Telomere shortening may be a link for poor outcomes in older vulnerable patients, as this is associated with both MCV variation and ageing-related all-cause mortality [43, 44]. Association of RDW increase with mortality was slightly lower, when NEWS2 was added as a potential confounder, which supports that elevated RDW is reflecting both short-, and long-term clinical deterioration.

RDW is a readily available biomarker for most ED patients. In clinical practice, RDW may be overlooked as a marker when clinical state and risks are assessed. Including RDW in patient assessment could lead to better high-risk feature identification, better targeting of further diagnostic work-up, effective interventions, and individualized advanced care planning.

Current risk-assessment methods, including ED triage systems, have limited performance, especially in older adults [45–47]. Machine-learning systems are promising tools for objective and more accurate risk assessment in emergency care, and may help in identifying patients who would benefit from targeted interventions [48–50]. In this study we have considered RDW as a general predictor. As higher RDW predicts poor outcomes in numerous different conditions, RDW may be associated with other predictive biomarkers in different specific conditions. Independence of RDW in multivariable predictive models could be studied preferably with machine learning methods with large data sets, as many predictive variables, including haemoglobin, white blood cell, and platelet counts, may have nonlinear associations.

Kim et al. stated in their article that RDW value should be included in risk stratification strategies for hospitalized older patients [29]. Based on earlier studies and our results, we agree with those authors and suggest that RDW should also be included in risk stratification of frail patients in emergency departments and hospital wards. Older patients often have non-specific presentations in the ED, and vital signs are less reliable for detecting early clinical deterioration in older patients [51–53]. Therefore, older patients, with or without frailty, could be one patient group that would benefit in particular from more comprehensive deep-learning risk-assessment methods. It may be favourable to include RDW among other variables when such artificial intelligence models are studied.

The strengths of this study include the prospectively collected patient data from a clinical setting, that is representative for the frail older ED patient population. Frailty status was assessed systematically with the CFS during ED admission. Baseline and outcome data were available for all patients included.

The study has some limitations. The analysis of this study was based on data collected in a previous prospective study. In this study, 82% of patients who met the eligibility criteria, had an RDW value available. The included patients had higher mortality than patients who had no laboratory testing, or only point-of-care testing available. Chief complaints or acute disease severity were not included in the analyses, but we assume that those patients without blood tests taken were more likely visiting the ED for simple, low-acuity complaints, and therefore the results may not be representative for low-acuity patients.

## Conclusion

Higher RDW values were significantly associated with increased 30-day mortality in frail older adults in the ED. In this study, RDW was independent of frailty state as a risk predictor. RDW is a readily available parameter for most ED patients who have laboratory tests. It might be beneficial to include RDW in risk stratification of older frail ED patients in order to identify patients at high risk of adverse outcomes who could benefit from further diagnostic assessment, targeted interventions, and care planning.

## Data Availability

The data are not publicly available or available for sharing due to national juridical restrictions. However, further description or analysis of data are available from the authors upon reasonable request. For inquiries, please contact the corresponding author.

## Abbreviations

RDW: Red cell distribution width
MCV: Mean corpuscular volume (of red blood cells)
ED: Emergency department
CFS: Clinical Frailty Scale
IQR: Interquartile range
OR: Odds ratio
CI: Confidence interval
NEWS2: National Early Warning Score 2
